# Associations of emotional social support, depressive symptoms, chronic stress, and anxiety with hard cardiovascular disease events in the United States: the multi-ethnic study of atherosclerosis (MESA)

**DOI:** 10.1186/s12872-023-03195-x

**Published:** 2023-05-04

**Authors:** Seyed Mohammad Riahi, Ahmad Yousefi, Farhad Saeedi, Seth Shay Martin

**Affiliations:** 1 Cardiovascular Diseases Research Center, Department of Epidemiology and Biostatistics, School of Medicine, Birjand University of Medical Sciences, Birjand, Iran; 2 PhD in Clinical Psychology, Department of Clinical Psychology, School of Medicine, Shahid Beheshti University of Medical Sciences, Tehran, Iran; 3 Student Research Committee, Birjand University of Medical Sciences, Birjand, Iran; 4 Cardiovascular Diseases Research Center, Birjand University of Medical Sciences, Birjand, Iran; 5 Johns Hopkins Ciccarone Center for the Prevention of Cardiovascular Disease, Baltimore, MD USA; 6 Division of Cardiology, Johns Hopkins University School of Medicine, Baltimore, MD USA

**Keywords:** Depressive symptoms, Anxiety, Chronic Burden Scale, Emotional social support, Cardiovascular Disease

## Abstract

**Background:**

Cardiovascular diseases (CVDs) are a major cause of morbidity and mortality around the globe and psychosocial factors are not sufficiently understood.

**Aim:**

In the current study, we aimed to evaluate the role of different psychosocial factors including depressive symptoms, chronic stress, anxiety, and emotional social support (ESS) on the incidence of hard CVD (HCVD).

**Methods:**

We examined the association of psychosocial factors and HCVD incidence amongst 6,779 participants in the Multi-Ethnic Study of Atherosclerosis (MESA). Using physician reviewers’ adjudication of CVD events incident, depressive symptoms, chronic stress, anxiety, emotional social support scores were measured by validated scales. We used Cox proportional Hazards (PH) models with psychosocial factors in several of the following approaches: (1) Continuous; (2) categorical; and (3) spline approach. No violation of the PH was found. The model with the lowest AIC value was chosen.

**Results:**

Over an 8.46-year median follow-up period, 370 participants experienced HCVD. There was not a statistically significant association between anxiety and HCVD (95%CI) for the highest versus the lowest category [HR = 1.51 (0.80–2.86)]. Each one point higher score for chronic stress (HR, 1.18; 95% CI, 1.08–1.29) and depressive symptoms (HR, 1.02; 95% CI, 1.01–1.03) was associated with a higher risk of HCVD in separate models. In contrary, emotional social support (HR, 0.98; 95% CI, 0.96–0.99) was linked with a lower risk of HCVD.

**Conclusions:**

Higher levels of chronic stress is associated with greater risk of incident HCVD whereas ESS has a protective association.

## Introduction

Cardiovascular diseases (CVDs) are a major clinical and public health burden and globally, CVD is a principal reason of death. World Health Organization projected 17.7 million CVDs death in 2015, demonstrating 31% of all worldwide mortalities. Stroke was responsible for approximately 6.7 million of these fatalities, whereas coronary heart disease was responsible for 7.4 million (CHD) [1]. Lack of physical activity, high blood pressure, obesity, smoking, diabetes mellitus, poor diet, high blood cholesterol, and excessive alcohol drinking are the traditional CVD risk factors [2, 3].

In the recent decade, the significance of psychosocial factors has been highlighted in the emergence of CVD. A growing body of studies have recognized associations between psychosocial characteristics and CVD incidence and mortality [4, 5]. In observational studies, it has been determined that high levels of anxiety and depression were linked with the incidence of CHD [6]. The annual prevalence of depression in the US cardiovascular patients was 9.3% in 2007 [7], whereas in US population it was 7% [8]. In another study, people with poor social support were at higher risk of premature development and mortality from coronary artery disease (CAD) [9]. Several studies have indicated that stressful conditions in family life and at work for a long time increase CAD risk, especially chronic stress at work forecasts premature CAD incident in men [10–12]. The INTERHEART study indicated that a group of risk factors of psychosocial disorders (i.e. stress in family life or at work, depression, and social deprivation) is related to higher risk for myocardial infarction [13]. Furthermore, studies have shown individuals with depression, anxiety, and suppressed anger had significant higher heart rates (HR) and reduction in HR variability that may have been resulted in the increased cardiac morbidity rate [14, 15]. Increased HR and/or reduced HR variability have characterized as an important predictor for incidence of CHD [16]. Camacho et al. sought to explore the associations of atherosclerosis and antidepressant using Multi Ethnic Study of Atherosclerosis (MESA) and found no statistically significant associations [17]. Based on an study utilizing UK Biobank and Emerging Risk Factors Collaboration including more 560,000 participants, depressive symptoms can increase the risk of CVDs [18]. The results of a multicenter study with a mean follow up of 9.3 years that included more than 145,000 participants from rural and urban communities showed that depressive symptoms increased the risk of CVD [19]. In a meta-analysis study including 323,709 participants, it was found that hazard ratio for association of depression with myocardial infarction and coronary death were 1.31 (95% CI, 1.09–1.57) and 1.36 (95% CI, 1.14–1.63), respectively [20]. Meng et al. conducted a cohort study that included more than 500,000 individuals and found that the hazard ratio for association of depression and all-cause mortality of CVD was 1.32 [95% CI, 1.20–1.46] [21]. In another cohort study including more than 12,000 individuals, Li et al. found that loneliness (adjusted hazard ratio, 1.21; 95% CI, 1.02–1.44) and restless sleep (adjusted hazard ratio, 1.21; 95% CI, 1.06–1.39) are two of depression symptoms that were significantly associated with CVD incidence [22]. Moreover, depression and anxiety could impact the cardiac rehabilitation [23]. Using English Longitudinal Study of Ageing (ELSA), Poole et al. found that depressive symptoms could predict the incidence of CHD (hazard ratio 1.11, 95% CI 1.04–1.20) [24].

In this study we sought to determine the association of various psychosocial factors such as chronic stress burden, depressive symptoms, anxiety, and emotional social support with the incidence of HCVD in a longitudinal manner.

## Methods

### Study Design and participants

The Multi Ethnic Study of Atherosclerosis (MESA) is a longitudinal cohort study that examines risk factors for clinical and subclinical CVDs in different racial/ethnic groups [25]. The number of participants in this study was 6,814 with a range of 45–84 years old who were enrolled from 6 major centers in the USA (St. Paul, MN; New York City, NY; Forsyth County, NC; Los Angeles, CA; Chicago, IL; Baltimore, MD). Samples included 47.2% males and 52.8% females, 38.6% Caucasian-Americans, 11.8% Chinese–Americans, 27.7% African–American, and 21.9% Hispanic-Americans and all of them were without any CVDs at enrollment to the study. In total, six exams with comparable procedures have been conducted (July 2000 to March 2018). We excluded participants who had missing data on all psychosocial indices as well as the outcome; 6,779 contributors were qualified for evaluation of chronic stress burden, symptoms of depression, anxiety as well as emotional social support. The blood samples were drawn from participants and sent to central laboratory at University of Minnesota and University of Vermont for biochemical assay, DNA extraction, and storage. White cells were also prepared for cryo-preservation to be stored for cell line generation in the future. Exposure variables were assessed by questionnaires in order to gather demographic information.

### Psychosocial indices

Standardized questionnaires were used according to the participants’ preferred language (Chinese, Spanish, or English) to evaluate psychosocial risk factors. Symptoms of depression were determined by utilizing the Center for Epidemiology Studies Depression (CES-D) Scale that contains 20-item and evaluates interactions with others, somatic complaints, mood, and motor functioning (range, 0–60) [26]. We generated five categories (i.e. 0–2, 3–5, 6–10, 11–15 and ≥ 16) for CES-D according to the distribution of score in estimated quartiles. Top quartile was divided into two categories, hence, the top category included 12.87% of individuals with a value ≥ 16. Chronic stress was measured by Chronic Burden Scale (CBS) Scale which evaluates stress based on severity and presence in 5 situations including job, health problems of close others, one’s own health problems, relationships, finances (ranging from 0 to 5) [27]; three stress categories were generated according to scores of 0, 1, and ≥ 2. Anxiety were examined by using Spielberger Trait Anxiety scale [28]. The Spielberger Trait Anxiety questionnaire is a ten-item scale that evaluates feel stress, worry, and discomfort in relatively enduring disposition (range, 10–40); four categories were generated according to the scores of 10–12, 13–14, 15–18, and ≥ 19. Emotional social support was evaluated using emotional social support index, that involves 6 items and evaluate availability of emotional social support (ranging from 6 to 30). Three categories were generated based on scores of Moderate (score < 18), high (score 19–24) or very high (score ≥ 25) levels of ESS [29, 30]. Investigation forms of MESA are accessible at http://www.mesa-nhlbi.org/ex1forms.aspx.

### Covariates

Socio-demographic characteristics for instance gender, age, race, and education were included. Behavioral factors such as total intentional exercise in met-min/week [31], smoking status (current smoker, former/never), and alcohol status (current drinker, former/never) were included. CVD risk factors including family history of heart attack (Parents/siblings/children), current aspirin use, hypertension, systolic blood pressure (SBP), antihypertensive medication use, triglycerides (TG), high-density lipoprotein cholesterol (HDL-C), low-density lipoprotein cholesterol (LDL-C), height (cm), body mass index (BMI) and diabetes mellitus status determined by criteria of American Diabetes Association and measuring fasting glucose [32] were also included. Additional covariates at baseline were C-reactive protein (CRP), interleukin-6 (IL-6), and fibrinogen measured using fasting blood specimens through standard assays.

### Outcome

The primary outcome of this study was hard CVD (which in this article we refer to them as HCVD) determined CVD incidence as CHD (definite CHD death, definite and likely MI, and resuscitated cardiac arrest), other atherosclerotic CVD death, or stroke (fatal or nonfatal). CVD or CHD death classified by reviewers as absent or present in accordance with hospital records and consultations with relatives. Definite CHD death was determined as a chest pain within the 72 h before death, MI within 28 days of death, or a CHD history with the absence of a recognized noncardiac or non- atherosclerotic cause of death. Myocardial infarction was categorized by Reviewers as absent, likely, or definite mainly according to the mixtures of electrocardiographic findings, symptoms, and biomarkers level (i.e. troponins or creatine kinase myocardial band). Neurologists evaluated and categorized a stroke as present if there was a focal neurologic impairment that continued for 24 h or until death, as well as a clinically significant damage on brain imaging and no nonvascular etiology. The follow-up duration of this study was from the baseline until the first CVD event, death, loss to follow-up, or February 2012, whichever came first.

The follow up period of this study for incident HCVD events was 8.46 years. A telephone interviewer, at period of 9 to 12 months, called each individual to investigate about cardiovascular outpatient diagnoses, interim hospital admissions, and deaths. In order to validate medical records for outpatient cardiovascular diagnoses, self-reported diagnoses, and hospitalizations, as well as copies of all death certificates was requested. In addition, relative interviews was conducted for cardiovascular deaths occurred out-of-hospital. If there were any differences after the first adjudication and evaluation, a comprehensive morbidity and mortality review committee made the final categorization.

### Statistical analysis

We calculated the descriptive statistics for all variables at baseline. We applied Cox proportional.

Hazards models to determine the impacts of interested variables on the hazard of HCVD events for each participant, person-year was calculated from baseline until the occurrence of a HCVD event, death, loss to follow-up, or non-occurrence of a HCVD event till February 22, 2012. Psychosocial factors were categorized as indicated in Table 1. The proportional hazards (PH) assumption was assessed by testing the interactions between interested independent variables and time and also checking the charts of the scaled Schoenfeld remaining versus rank of time and no violation of the PH assumption was found.

**Table 1:** Adjusted hazard ratios (95% confidence intervals) for categorical psychosocial factors and risk of hard CVD incidence: The Multi-Ethnic Study of Atherosclerosis (2000–2012)

	Category 1	Category 2	Category 3	Category 4	Category5	P _trend_
Depressive symptoms range	0–2	3–5	6–10	11–15	≥ 16	
Number of Event	97	76	96	43	55	-
Number of participants	1829	1549	1707	816	872	-
Model1†	1(reference)	0.90(0.66–1.22)	1.12(0.83–1.49)	1.01(0.69–1.47)	1.32(0.92–1.90)	0.09
Model2††	1(reference)	0.87(0.63–1.21)	1.18(0.86–1.59)	1.00(0.67–1.47)	1.30(0.89–1.90)	0.14
Chronic stress range	0	1	≥ 2			
Number of Event	108	129	130	-	-	-
Number of participants	2308	2108	2332	-	-	-
Model1†	1(reference)	1.37(1.05–1.79)	1.51(1.16–1.96)	-	-	0.001
Model2††	1(reference)	1.35(1.02–1.79)	1.53(1.15–2.04)	-	-	0.003
Anxiety range	10–12	13–14	15–18	≥ 19		
Number of Event	106	61	95	104	-	-
Number of participants	1766	1235	1957	1790	-	-
Model1†	1(reference)	0.94(0.68–1.31)	0.99(0.74–1.37)	1.25(0.93–1.67)	-	0.17
Model2††	1(reference)	0.66(0.29–1.49)	0.87(0.42–1.77)	1.51(0.80–2.86)	-	0.17
Emotional social support range	6–18	19–24	≥ 25			
Number of Event	61	119	185	-	-	-
Number of participants	1022	2029	3712	-	-	-
Model1†	1(reference)	0.92(0.67–1.26)	0.70(0.52–0.95)	-	-	0.009
Model2††	1(reference)	0.89(0.64–1.24)	0.69(0.51–0.94)	-	-	0.009

†Model 1: adjusted for age, sex, race, education, and family history of heart attack

††Model 2: adjusted for model 1 plus smoking, alcohol status, diabetes status, current aspirin use, hypertension medication use, and total intentional exercise, LDL-C, HDL-C, systolic blood pressure, triglycerides, and BMI.

Higher scores on the psychosocial factors show more severe symptoms (Except emotional social support)

Our analyses were conducted in several models. Initial models were controlled for sex, race, age, education, and family history of heart attack. In model 2 we added smoking, status of alcohol drinking, status of diabetes, current aspirin use, hypertension medication use, and total intentional exercise, LDL-C, HDL-C, systolic blood pressure, triglycerides, and BMI. Furthermore, model 3 controlled for fibrinogen, IL-6, and C-reactive protein (CRP). TG and CRP were log-transformed in all analyses to account for skewness. Covariate Age in analyses of Chronic Stress burden on HCVD was considered as a cubic equation.

In modeling process, we included each of the psychosocial factors in several following approaches: (1) continuous approach in which all those were entered to the models as continuous variable; (2) categorical approach in which all those were categorized base on Sect. 2.2. (3) spline approach in which we conducted a piecewise cox regression after using linear and restricted cubic spline, that was run via mkspline command in stata, in order to consider non-linear effects of psychosocial factors on risk of incident HCVD. In this setting, we set the psychosocial variables to a given reference (for example, 16 for CES-D; 0 for CBS; 10 for anxiety; 30 for ESS). Then, in those references the relative hazard would be 1. Based on the above approaches, a final model for each psychosocial factor was selected according to the Akaike information criterion (AIC). Given a collection of potential models fitting the data, the model with the lowest AIC score was considered the ideal one. Owing to AIC values, the best approach in our modeling for CES-D, CBS, and ESS was continuous approach, whereas the best approach for anxiety was categorical. Based on the AIC value, a spline approach was not suitable for any of the psychosocial factors. The conclusion of this study is based on Cox models but more models were provided for more evidence.

Multiplicative interactions between each of psychosocial indices and sex, race, and age were tested by including cross-product terms in all models. Parametric test (T-test) and non- parametric test (chi square) used for analyzing the baseline characteristics. All analyses were performed utilizing Stata 14. A p value < 0.05 was considered as statistically significance.

## Results

### Baseline characteristics

A total of 370 new cases of CVD were diagnosed during a follow-up period of 51,752 person-years. Mean period of follow-up for participants was 8.46 years (range, 0.01–10.92). The HCVD incidence rate was 7.1 cases per 1000 person-years (95% CI 6.4–7.9). The total average age at the baseline was 62.2 ± 10.2 years, and 47.2% of the sample was male. At baseline, the overall mean scores were 7.6 ± 7.6 for symptoms of depression, 1.2 ± 1.2 for Chronic Stress, 24.1 ± 5.2 for emotional social support, and 15.9 ± 4.5 for anxiety. There were 2614 (38.6%) Caucasian-Americans, 1879 (27.7%) African-Americans, 1485 (22.9%) Hispanic-Americans, and 801 (11.8%) Chinese-Americans. The baseline characteristics of MESA contributors by HCVD status are represented in Table 2. In general, those who experienced HCVD through follow-up were older, had lower HDL-C, had fewer MET-minutes/week of total intentional exercise, had higher systolic blood pressure, and had higher TG at baseline in comparison to those who did not experience HCVD during follow-up. Those who had HCVD throughout follow-up were also more likely to be current or former smokers and to be using hypertension medication at baseline than those who did not experience HCVD. As mentioned above, psychosocial factors were included into the models by three following approaches: (1) continuous; (2) categorical (3) spline.

**Table 2:** Participant characteristics, baseline: Multi-Ethnic Study of Atherosclerosis, 2000 to 2002

	All participant	HCVD	No HCVD	P. value*
N total	6779	370	6409	
**N (%)**				
Age	62.2 ± 10.2	68.0 ± 9.7	61.8 ± 10.2	< 0.001
Gender				
Male	3197(47.2)	220(59.5)	2977(46.5)	< 0.001
Female	3582(52.8)	150(40.5)	3432(53.5)	
Education				
Less than diploma	1215(17.0)	85(23.0)	1130(17.7)	< 0.001
Diploma or some college	2814(41.6)	169(45.8)	2645(41.4)	
College degree or higher	2729(40.4)	115(31.2)	2614(40.9)	
Race				
Caucasian -Americans	2614(38.6)	156(42.2)	2458(38.3)	0.003
Chinese -Americans	801(11.8)	22(5.9)	779(12.2)	
African -American	1879(27.7)	102(27.6)	1777(27.7)	
Hispanic -Americans	1485(22.9)	90(24.3)	1395(21.8)	
Alcohol status				
Never	1382(20.5)	74(20.1)	1308(20.6)	0.004
Former	1609(23.9)	114(31.0)	1495(23.5)	
Current	3739(55.6)	180(48.9)	3559(55.9)	
Smoking status				
Never	3400(50.3)	172(46.6)	3228(50.5)	0.003
Former	2475(36.6)	127(34.4)	2348(36.7)	
Current	884(13.1)	70(19.0)	814(12.8)	
Diabetes				
Normal	4972(73.6)	226(61.3)	4746(74.3)	< 0.001
Impaired fasting glucose	933(13.8)	54(14.6)	879(13.8)	
Untreated diabetes mellitus	179(2.6)	10(2.7)	169(2.6)	
Treated diabetes mellitus	671(10.0)	79(21.4)	5928(9.3)	
Current aspirin use(at least 3 days/wk)				
No	5202(80.0)	270(76.5)	4932(80.3)	0.07
Yes	1201(20.0)	83(23.5)	1208(19.7)	
Hypertension medication use				
No	4250(62.7)	175(47.4)	4075(63.60)	< 0.001
Yes	2526(37.3)	194(52.6)	2332(36.4)	
Family history of heart attack				
No	3639(57.3)	162(47.2)	3477(57.8)	< 0.001
Yes	2722(42.8)	181(52.8)	2541(42.2)	
Mean(SD)				
Age, y	62.2(10.2)	68.0(9.7)	61.8(10.2)	< 0.001
Height, cm	166.4(10.0)	166.6(10.4)	166.3(9.9)	0.32
Total intentional exercise (MET-min/wk)	1556.2(2344.0)	1260.7(1808.8)	1573.2(2370.2)	0.006
LDL-C, mg/dl	117.2(31.5)	118.7(31.7)	117.1(31.5)	0.34
HDL-C, mg/dl	51.0(14.8)	47.4(13.7)	51.2(14.9)	< 0.001
Systolic blood pressure, mmHg	126.6(21.5)	137.8(23.0)	125.9(21.2)	< 0.001
Triglycerides, mg/dl	131.5(88.8)	143.6(81.4)	130.8(89.2)	< 0.001
BMI,Kg/m2	28.3(5.4)	28.6(5.1)	28.3(5.4)	0.17
C-Reactive Protein, mg/L	3.8(5.8)	4.3(6.0)	3.7(5.8)	0.03
Fibrinogen, mg/dL	346.7(73.9)	369.4(82.5)	345.4(73.2)	< 0.001
Interleukine-6	1.6(1.2)	1.9(1.4)	1.5(1.2)	< 0.001
Psychosocial factor				
CES-D (Range,0–60)	7.6(7.6)	8.1(8.1)	7.5(7.5)	0.06
CBS (Range,0–5)	1.2(1.2)	1.3(1.2)	1.2(1.2)	0.15
ESS (Range,6–30)	24.1(5.2)	23.8(5.5)	24.2(5.3)	0.36
Anxiety (Range,10–40)	15.9(4.5)	15.8(4.6)	15.9(4.5)	0.62

BMI indicates body mass index; HDL-c, high-density lipoprotein- cholesterol; CES-D, Center for Epidemiologic Studies Depression Scale; MET, metabolic equivalent of task; LDL-c, high-density lipoprotein-cholesterol; SBP, systolic blood pressure; and CVD, cardiovascular disease; CBS, Chronic Burden Scale; ESS, Emotional Social Support

*P values from t tests or χ2 tests or, as appropriate, are for comparisons of those who did or did not experience a CVD during follow-up

### Continuous and categorical approach

**Fig. 1:**
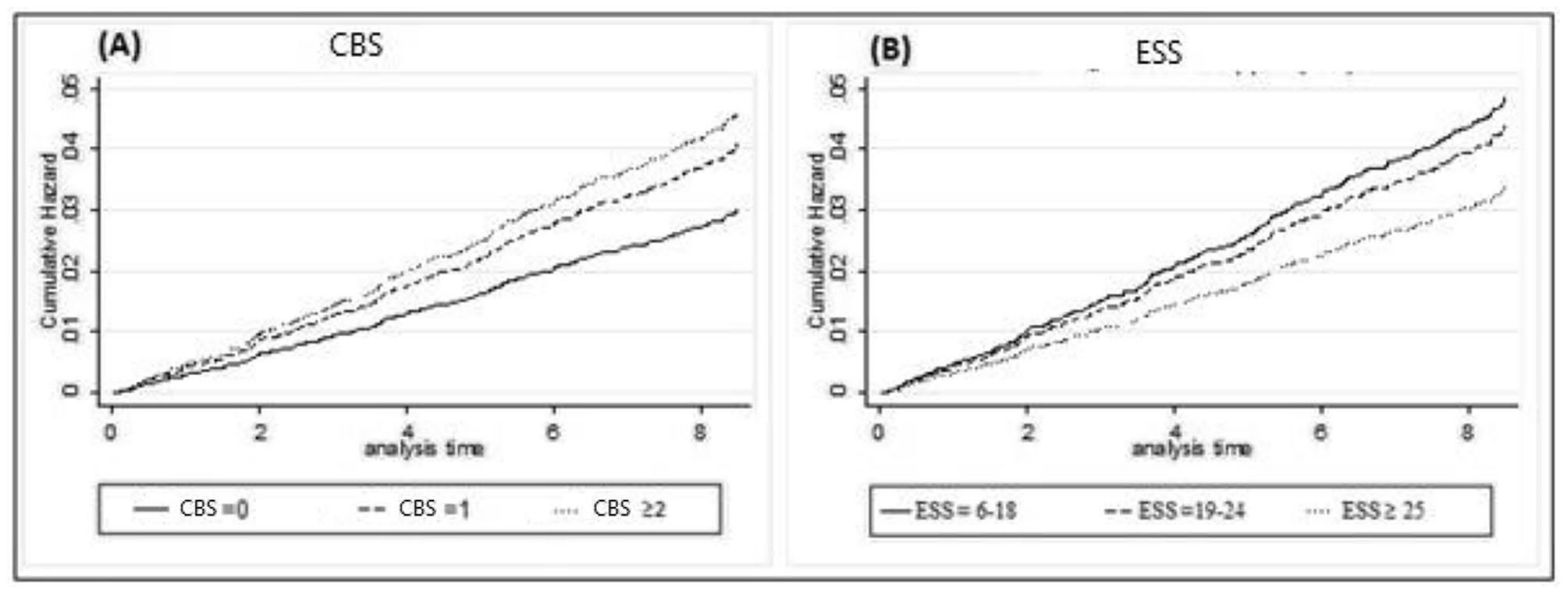
Cox proportional cumulative hazard for HCVD stratified by CBS (panel A) and ESS (panel B) status, MESA, 2000 to 2002. Adjusted for age, sex, race, education, family history of heart attack, smoking, alcohol status, diabetes status, current aspirin use, hypertension medication use, and total intentional exercise, LDL-C, HDL-C, systolic blood pressure, triglycerides, and BMI

In continuous approach, controlling for education, sex, race, age and family history of heart attack, each 1 point higher score for chronic stress (HR, 1.18; 95% CI, 1.08–1.29) and depressive symptoms (HR, 1.02; 95% CI, 1.01–1.03) was associated with a higher risk of HCVD in different models. Conversely, ESS (HR, 0.98; 95% CI, 0.96–0.99) was related with a lower risk of HCVD. Associations remained significant with additional adjustment (chronic stress: HR, 1.17; 95% CI, 1.06–1.29; emotional social support: HR, 0.98; 95% CI, 0.96– 0.99). In general, depressive symptoms and anxiety were not considerably related to risk of HCVD (P > 0.05). Owing to categorical approach, Table 1 indicates hazard ratios and 95% CI for each of the psychosocial factors related to risk of HCVD incidence. In model 1, which was adjusted for, race, sex, age education, and family history of heart attack, we observed that gradients of higher risk for chronic stress and ESS were statistically significant; the trend for depressive symptoms and anxiety was not significant but relations were in the anticipated direction. As shown in Table 1, individuals in the top group (maximum scores) had a 1.5-fold greater risk of HCVD during follow-up, compared with the lowest category [HR = 1.51 (95% CI: 1.16–1.96)]. In contrast, individuals in the highest category for ESS had lower risk of CVD throughout follow-up, compared with the lowest category [HR = 0.70 (95% CI: 0.52–0.95)]. In model 2, which controlled for model 1 plus smoking, alcohol status, diabetes status, current aspirin use, hypertension medication use, and total intentional exercise, LDL-C, HDL-C, systolic blood pressure, triglycerides, and BMI, HR for chronic stress and ESS were remained significant after further adjustment. The addition of inflammatory markers (i.e. CRP, IL6, and fibrinogen) and anti-depressant use in model 2 did not change the estimates (data are not shown). Cumulative hazard functions which were adjusted by the interested covariates in model 2 were plotted and given in Fig. 1. It demonstrates higher cumulative hazard for HCVD in higher levels of chronic stress and lower levels of ESS.

### Spline approach

**Fig. 2:**
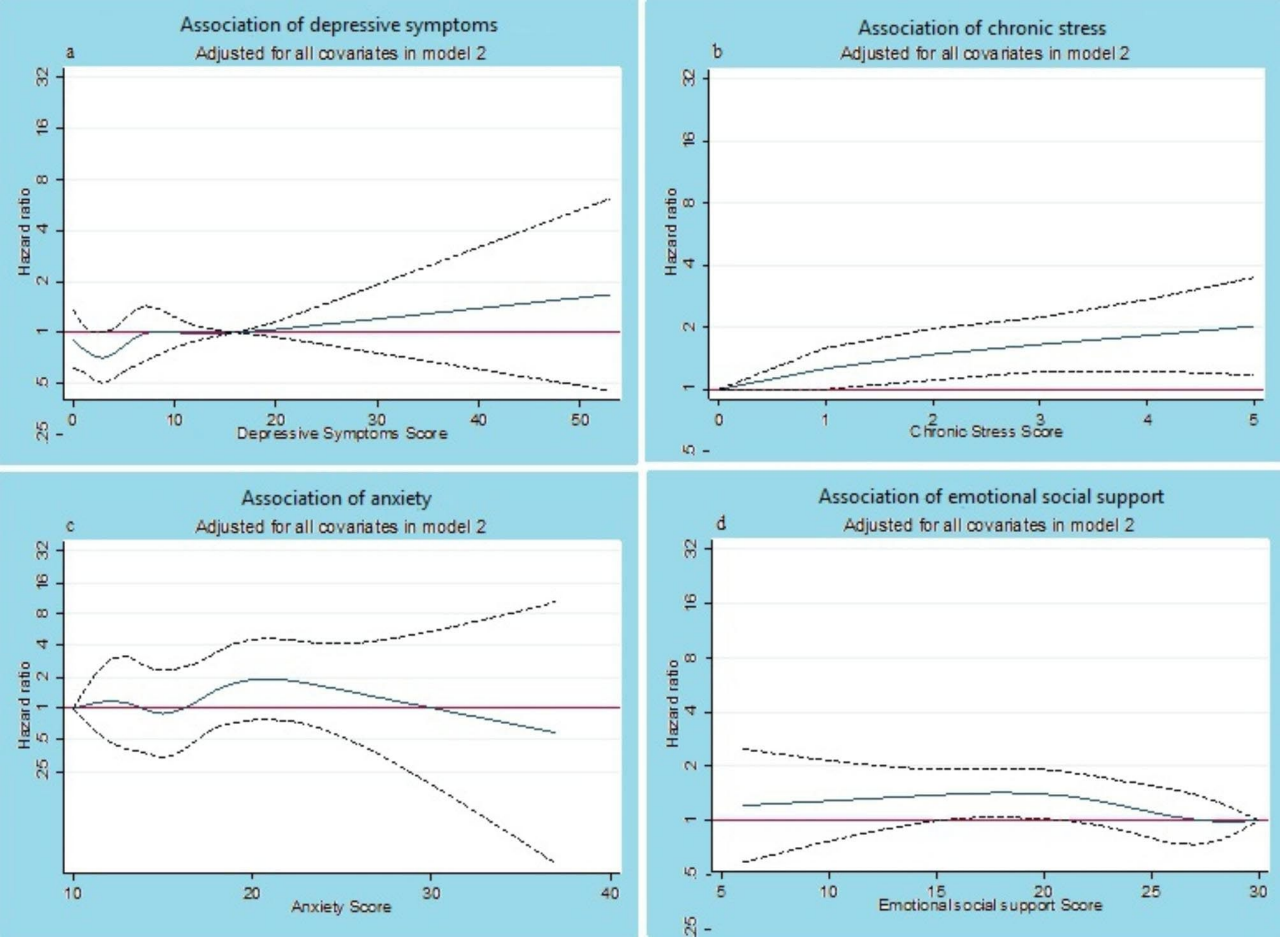
Piecewise cox regression hazard ratios (solid line) and 95% CI (dash lines) for HCVD stratified by psychological factor, MESA, 2000 to 2002. Adjusted for age, sex, and race, and education, family history of heart attack, smoking, alcohol status, diabetes status, current aspirin use, hypertension medication use, and total intentional exercise, LDL-C, HDL-C, systolic blood pressure, triglyceride, and BMI. Reference level in spline model was 16 for CES-D (a); 0 for CBS (b); 10 for anxiety(c); 30 for ESS (d). Hazard ratio line equal to 1 shows not-significant level

According to spline approach, Fig. 2 shows results of piecewise cox regression hazard ratios and 95% CI, controlled for all variables in model 2, for each psychosocial factor relating to risk of incident HCVD. As shown in this figure, individuals in the highest level of chronic stress were at 2-fold higher risk of HCVD throughout follow-up, compared with the lowest scoring level (i.e. 0).The tendency for depressive symptoms was not noteworthy but associations were in the anticipated direction from cut-point 16 and more. In contrast, individuals in the lowest level of ESS had higher risk of HCVD during follow-up, compared with the highest scoring level (i.e. 30). Our findings did not show any given pattern for anxiety relating to risk of incident HCVD. In order to select the best approach for each of psychosocial factors, we calculated AIC value for each of them so that the model which had the minimum AIC value was the ideal one. According AIC vales, the continuous approach was acceptable for depressive symptoms, chronic stress, and ESS factors and categorical approach for anxiety factor. AIC vales in all spline models was higher than two other approach and they were not therefore preferred model. No interactions were found between age, sex and race with the interested psychosocial indices (all p-value > 0.1; data are not shown).

## Discussion

In this study, higher levels of chronic stress were associated with a higher risk of HCVD. There was a comparable trend for depressive symptoms, although the observed effect was non-significant in categorical analyses, most likely due to observing few events. In addition, we observed that higher levels of emotional social support had a protective association with the incidence of HCVD. The associations between psychosocial factors and incidence of HCVD were comparatively unchanged following controlling for additional known risk factors of HCVD in our main analyses, utilizing the ones being evaluated at the first examination. This study highlights the potential important role of psychosocial factors in assessing the risk of HCVD.

This study was best designed to observe associations, but in exploring the possibility of a causal relationship, the pathways by which psychosocial factors might lead to higher risk of HCVD warrants consideration. Psychosocial factors and CVD may connect through three pathways: unhealthy life style, pathophysiologic, and third underlying factors (alternative mechanisms). Psychosocial factors including depressed mood, excessive stress in life and anxiety by increasing unhealthy behaviors (including overeating, smoking, higher alcohol drinking, high levels of physical inactivity) and by reducing the adherence to specific treatments for heart diseases, contribute to an elevated risk of CVD. The Netherlands Study of Depression and Anxiety (NESDA) indicated that depressed patients achieved high scores in all unhealthy life style indices [33]. Bonnet et al. concluded that anxiety and depression were associated with high levels of physical inactivity, unhealthy diet and smoking habits in both sexes [34]. Using MESA dataset, Kershaw et al. investigated the association of chronic neighborhood stressors with incident of coronary heart diseases and found that individuals in high and medium category of stressors had 65% and 49% higher risk of coronary heart diseases incident, compared to low risk category [35]. Everson-Rose et al. explored the association of hostility, chronic stress, and depressive symptoms with stroke using MESA database and showed that HR of 2.22, 1.59, and 1.86 which all were statistically significant for hostility, chronic stress, and depressive symptoms, respectively [36]. Moreover, researchers have shown that depressed mood creates difficulty in treating by intrusive in compliance with medical advice and treatment regimens [37, 38]. The present study shows that individuals who experience higher levels of chronic stress and symptoms of depression (in the continuous approach) tend to carry higher risk for HCVD. However, our study did not assess precise pathways associated with causal mechanism.

Psychosocial features by common pathophysiological pathways link to HCVD outcomes. One of the most trustworthy discoveries of biological psychiatry is hyperactivity of the hypothalamic-pituitary-adrenal axis (HPA-axis or HTPA axis) in depression and anxiety. As such, negative emotions and stress trigger the HPA-axis, resulting in alterations in glucocorticoids and increasing in circulating catecholamine; which impact platelet activation and endothelial dysfunction and have neuroendocrine, metabolic, and immunologic effects [39, 40]. Some psychosocial disorders like depression and anxiety by autonomic dysregulation are involved in cardiovascular somatic symptoms such as heart rate variability, especially when the person is under a severe stress [15, 41].

The psychosocial indices measured in current study were associated with higher inflammatory markers (i.e. CRP, IL6, and fibrinogen) separately, which may relate to increased risk of CVD risk [42, 43]. Yet, controlling for these markers did not alter observed associations. In this study, only baseline measurements of these markers were available, but it supplies some evidence for an inflammatory pathway. Our results show that people who experience higher levels of ESS (in continuous as well as categorical approach) could have lower risk of HCVD. Various studies have shown relations between chronic psychosocial stress and shorter Leukocyte Telomere Length (LTL) [44, 45]. Furthermore, it seems that social support can reduce the severity of response to stressful conditions by acting as a buffer to appraisal of threats [46, 47]. Furthermore some investigations explain association between psychosocial factors and CVD outcomes through focus on the alternative mechanisms (i.e. childhood maltreatment, personality traits, genetic vulnerability) that increase the risk of both psychosocial conditions and CVD events [48–51].

### Limitations and strengths

First, the results of this study could not show the causality of psychosocial factors with CHD. Second, our study do not include the assessment of a history of psychiatric disorder and psychotropic medication use. Several noteworthy strengths also existed in this study including ascertainment of multiple psychosocial indices, a multi-ethnic representative population-based sample, using several statistical approach in analyses, and adjudicated HCVD outcomes. Further research with a higher power is necessary for future to reach more definitive results.

### Conclusions

This study indicates associations between higher risk of HCVD development and symptoms of depression, anxiety, chronic stress, and emotional social support, which were not elucidated by traditional CVD risk factors, subclinical atherosclerosis, or inflammatory markers. Higher levels of chronic stress and lower levels of ESS are associated with considerably higher risk of incident CVD. Although we found the association of depressive and anxiety symptoms with HCVD, these associations in the fully adjusted model were not significant. Given the aging population and increasing CVD burden, further studies are needed to address the importance and role of psychosocial factors in CVD.

## Data Availability

The data that support the findings of this study are available from BioLINCC but restrictions apply to the availability of these data, which were used under license for the current study, and so are not publicly available. Data are however available from the corresponding authors upon reasonable request and with permission of BioLINCC.

## Abbreviations

MESA: Multi-Ethnic Study of Atherosclerosis
HCVD: Hard Cardiovascular diseases
SBP: Systolic Blood Pressure
WHO: World Health Organization
CHD: Coronary Heart Disease
IL-6: Interleukin-6
CAD: Coronary artery disease
HR: Heart Rates
CES-D: Center for Epidemiology Studies Depression
ESS: Emotional Social Support
CBS: Chronic Burden Scale
HDL-C: High-Density Lipoprotein Cholesterol
TG: Triglycerides
CRP: C-reactive protein
LDL-C: Low-Density Lipoprotein Cholesterol
PH: Proportional Hazards
AIC: Akaike information criterion
HR: Hazard Ratio
LTL: Leukocyte Telomere Length
BMI: Body Mass Index
