# Correction to: Associations of emotional social support, depressive symptoms, chronic stress, and anxiety with hard cardiovascular disease events in the United States: the multi-ethnic study of atherosclerosis (MESA)

**DOI:** 10.1186/s12872-023-03298-5

**Published:** 2023-05-19

**Authors:** Seyed Mohammad Riahi, Ahmad Yousefi, Farhad Saeedi, Seth Shay Martin

**Affiliations:** 1 Cardiovascular Diseases Research Center, Department of Epidemiology and Biostatistics, School of Medicine, Birjand University of Medical Sciences, Birjand, Iran; 2 Department of Clinical Psychology, School of Medicine, Shahid Beheshti University of Medical Sciences, Tehran, Iran; 3 Student Research Committee, Birjand University of Medical Sciences, Birjand, Iran; 4 Cardiovascular Diseases Research Center, Birjand University of Medical Sciences, Birjand, Iran; 5 Johns Hopkins Ciccarone Center for the Prevention of Cardiovascular Disease, Baltimore, MD USA; 6 Division of Cardiology, Johns Hopkins University School of Medicine, Baltimore, MD USA

Following publication of the original article [1], the label of Part A in Fig. 1 has been updated.

The original article has been corrected.

